# Miscellany

**Published:** 1902-07

**Authors:** 


### Miscellany.

An Emergency Crown.—Grind the root down to the gum margin, measure the distance from the gum margin to the incisal edge of the adjacent tooth and the width of the space where the crown is missing, select the proper shade, and send to depot for a suitable facing, if you have not one at hand. While a facing is being selected enlarge the root-canal to the proper depth and adjust a post of such length that the lower incisors will not strike it as they articulate, and set back in the root far enough lingually so that it will not force the facing out of the line of the arch; flatten slightly the projecting end of the post and roughen the section to be embedded in the root; fill the root-canal with a large, soft, gutta-percha cone; heat the post, grasp the flattened end with a suitable pair of pliers, force it to its place in the root-canal, and trim away the surplus gutta-percha; slightly roughen with a coarse sand-paper disk the interproximal contact points of the adjacent teeth. The facing should have arrived by this time; grind it to fit properly, roughen the back on the facing with a carborundum wheel and bend down the pins so that they are at an angle of about forty-five degrees to it.

Dry the facing, the adjacent teeth, the post and root thoroughly, mix thickly some of Ames's quick-setting cement, coat the back of the facing and cover the end of the root and post with it, then force the facing into this mass of cement and hold it in position until the cement has set slightly; coat the cutting edge of the lower incisors and have the patient bite, then, opening the mouth, trim away the surplus cement, clean out the interproximal spaces, and contour as you please, but leave the cement adhering to the interproximal contact points to give greater security of retention. By this time the cement is quite thoroughly set and the patient may be dismissed.

The time consumed in this operation should not be more than a half-hour if you are within three blocks of a dental depot, and you may accomplish it in fifteen minutes if you have the facing at hand.—J. E. NYMAN, *Dental Review.*

---

PROCESS FOR PRODUCING GOLD-LIKE ALLOY FROM COPPER AND ANTIMONY.—This invention, patented in Germany, covers a metallic alloy, to take the place of gold, which, even if exposed for some time to the action of ammoniacal and acid vapors, does not oxidize nor lose it's gold color. It can be rolled and worked like gold, and has the appearance of genuine gold without containing the slightest admixture of that metal, besides being much cheaper than other precious and semi-precious metals, as well as the compounds and alloys used as substitutes for precious metals.

The alloy consists of copper and antimony in the approximate ratio of 100 to 6, and is produced by adding to molten copper, as soon as it has reached a certain degree of heat, the said percentage of antimony. When the antimony has likewise melted and entered into intimate union with the copper, some charcoal ashes, magnesium, and lime-spar are added to the mass while the latter is still in the crucible. Although the action of this material admixture of flux is not entirely explained, the alloy loses thereby a certain porosity otherwise present, and an exceedingly great density of the cast metal is obtained. It can now be rolled, wrought, hammered and soldered like gold, and when polished has the appearance of genuine gold, while being considerably firmer than the latter.—*Journal der Goldschmiedekunst, Scientific American.*

---

CONCEALED BICUSPID ATTACHMENT FOR BRIDGE.—Devitalize the bicuspid, and prepare canal as usual through an opening drilled in the centre of the sulcus of the tooth. Enlarge for the accommodation of 20-gauge wire (irido-platinum) and of good length. With small carborundum stone increase the depth and breadth of the sulcus until it becomes a wide, deep groove, particularly so on the side adjoining the space for the dummy. If the dummies are to be inserted on the mesial side of the tooth, then this aspect of the tooth must be ground flat and perpendicular, straight up and down, from the occlusal edge of the tooth to a point slightly under the cervical margin. Take impression of tooth in plaster, make a fusible metal die, filling up the impressions of the adjoining teeth with mouldine before pouring the metal. Swage 30-gauge pure gold over the mesial and occlusal aspects by driving the metal tooth into a transverse section into a block of soft pine wood. Punch hole for the 15-gauge irido-platinum post, place in position on the tooth with the post in the root-canal, fasten with Parr's wax, remove, invest, and tack post with 20-carat solder. Replace on the tooth and burnish and trim perfectly, allowing the plate to lap well over the edges. A good fit and the subsequent durability of the bridge depend upon close adaptation of the gold backing, which can only be secured by burnishing and thickening with 20-carat solder. Solder on dummy, contour and finish properly, and a strong and artistic bridge pier is the result. Set with cement and finish on the tooth with carborundum stone and sand-paper disks.—H. M. KIRKE, *Dental Register.*

---

PERCENTAGE SOLUTIONS.—The *Dental Office and Laboratory* gives the following from the *Pharmaceutical Journal:*

For all practical purposes, in dealing with single ounces, four and a half grains is near enough to form a one per cent. solution. With larger quantities it is, of course, obvious that a higher degree of accuracy may be obtained.

The above illustration shows the superiority of the metric system where there is a direct relationship between weight and volume, which is not the case with grains and minims. One minim does not represent the volume of one grain of distilled water, consequently a solution which contains one grain of a substance in one hundred minims is not a one per cent. solution. It should be observed that true percentage solutions contain a number of parts indicated in a hundred parts of a solution, the parts being all of one denomination. A one per cent. solution really indicates one grain in one hundred grains by weight. For dispensing purposes, parts per one hundred fluid parts are usually intended, although not distinctly stated, when percentage solutions are ordered. This would be equivalent to grains per hundred fluid grains, or 4.375 grains per fluidounce, which contains 437.5 fluid grains of water.

The symbol ℥ really should only be used to indicate the Troy ounce, containing 480 grains. Conveniently, however, it is employed to indicate the fluidounce containing 437.5 fluid grains.

[The above article is misleading. A grain is a grain whether of a liquid or a solid. There is no such term as "fluid grain."

Again, the gallon of apothecaries' fluid measure is the standard unit of liquid measure, the wine gallon. It contains 231 cubic inches and weighs 58372.1754 grains, or nearly eight and one-third pounds avoirdupois, of distilled water at 39.83° Fahrenheit, the barometer 30 inches. Divide this number of grains by 128, the number of ounces in a gallon, and the result is 456.03 grains, instead of 437.5 grains, the number of grains in an ounce avoirdupois weight; hence 456.03 grains is the basis upon which to estimate percentage solutions for fluidounces.—McClain.]

